# Adaptations of Natural Killer Cells to Self-MHC Class I

**DOI:** 10.3389/fimmu.2014.00349

**Published:** 2014-07-22

**Authors:** Stéphanie Bessoles, Camille Grandclément, Elisenda Alari-Pahissa, Jasmine Gehrig, Beena Jeevan-Raj, Werner Held

**Affiliations:** ^1^Department of Oncology, Ludwig Center for Cancer Research, University of Lausanne, Lausanne, Switzerland

**Keywords:** NK cells, MHC class I, education, licensing, Ly49 receptors

## Abstract

Natural Killer (NK) cells use germ line encoded receptors to detect diseased host cells. Despite the invariant recognition structures, NK cells have a significant ability to adapt to their surroundings, such as the presence or absence of MHC class I molecules. It has been assumed that this adaptation occurs during NK cell development, but recent findings show that mature NK cells can also adapt to the presence or absence of MHC class I molecules. Here, we summarize how NK cells adjust to changes in the expression of MHC class I molecules. We propose an extension of existing models, in which MHC class I recognition during NK cell development sequentially instructs and maintains NK cell function. The elucidation of the molecular basis of the two effects may identify ways to improve the fitness of NK cells and to prevent the loss of NK cell function due to persistent alterations in their environment.

## Development and Function of Conventional NK Cells

Natural killer (NK) cells, which mediate innate immunity to certain pathogens, are able to eliminate malignant cells and regulate innate and adaptive immune responses. These functions depend on a significant number of distinct germ-line encoded receptors. While a few of these receptors are specific for pathogens (1) or for pathogen-derived components expressed on infected host cells, the majority of NK cell receptors are specific for endogenous self-ligands. This type of receptor allows NK cells to respond to host cells whose expression of specific self-ligands deviates from normal.

Conventional NK cells arise from committed progenitors present in the bone marrow. The understanding of the transcriptional network guiding NK cell differentiation has significantly improved recently [for a review, see Ref. (2)]. Expression of the transcription factor Nfil3 (E4BP4) commits common lymphoid progenitors to the NK cell lineage (3). E4BP4 controls the expression of Eomes as well as Id2 (3) and Eomes and T-bet are essential for the development of conventional NK cells downstream of NK committed progenitors (4, 5). Immature NK cells undergo sequential differentiation, expansion, and maturation processes. They acquire a significant number of distinct activation and co-activation receptors, some of which depend on T-bet and Eomes (5). In addition, NK cells show variegated expression of inhibitory receptors, many of which are specific for MHC class I molecules. Furthermore, during their maturation in the bone marrow, NK cells directly acquire an effector program. Bone marrow NK cells constitutively express high levels of Perforin, Granzyme B, and IFNγ mRNAs that are, however, not translated into protein (6, 7). The trigger for the acquisition of this effector program is currently not known, but IL-17 signaling may play a role (8).

The execution of effector functions by peripheral NK cells is controlled at two additional levels. The translation of preformed Perforin and Granzyme B mRNA is dependent on NK cell priming by cytokines such as IL-15 (9). Of note, NK cells in human or wild mice display a primed phenotype, which likely reflects a more frequent exposure to pathogens. Finally, the exocytosis of lytic granules and the production and release of cytokines are tightly controlled by inhibitory and activating NK cell surface receptors. NK cells are stimulated by “non-self,” “stress induced self,” and/or “constitutive self” ligands on host cells. They are inhibited by ubiquitously expressed MHC class I molecules, which are recognized by inhibitory Ly49 (mouse), CD94/NKG2A (mouse human), or KIR family NK cell receptors (human). In general, an excess of activating over inhibitory signals triggers the production and release of effector molecules, which can lead to the death of the infected or transformed host cell (10). “Missing-self” recognition refers to NK cell-mediated responses to cells, which lack inhibitory MHC class I molecules.

## Adaptations of NK Cell to Their Environment

Despite the use of invariant germ-line encoded recognition receptors to detect diseased host cells, phenotypes and responses can significantly vary depending on the milieu that surrounds NK cells. Much of the diversity of human NK cells, which was recently estimated at 6,000–30,000 phenotypically distinct NK cell subsets, arises based on environmental variables, rather than genetic cues (11). The persistent exposure of NK cells to cell-bound stress ligands (12, 13) or viral ligands (14, 15) induced the downregulation of the cognate activation receptor and its non-responsiveness to re-stimulation. Dysfunction of activation receptors could be induced in mature NK cells and extended in some cases to activation receptors that had not been triggered [termed cross-tolerance induction (12)]. Removal of the activating ligand resulted in the restoration of receptor function. Similar effects were observed when NK cells were exposed to tumors *in vitro* and *in vivo* (12, 16). In addition to reduced function, persistent exposure to activating ligands was also associated with partial or almost complete loss of NK cells (15, 17). Chronic activation may thus affect, in a hierarchical manner, the function of cognate, followed by non-cognate activation receptors and eventually NK cell viability, similar to the exhaustion of CD8 T cells (18). The functional changes may also reflect an adaption of NK cells to their surroundings.

## NK Cell Education

**Figure 1:**
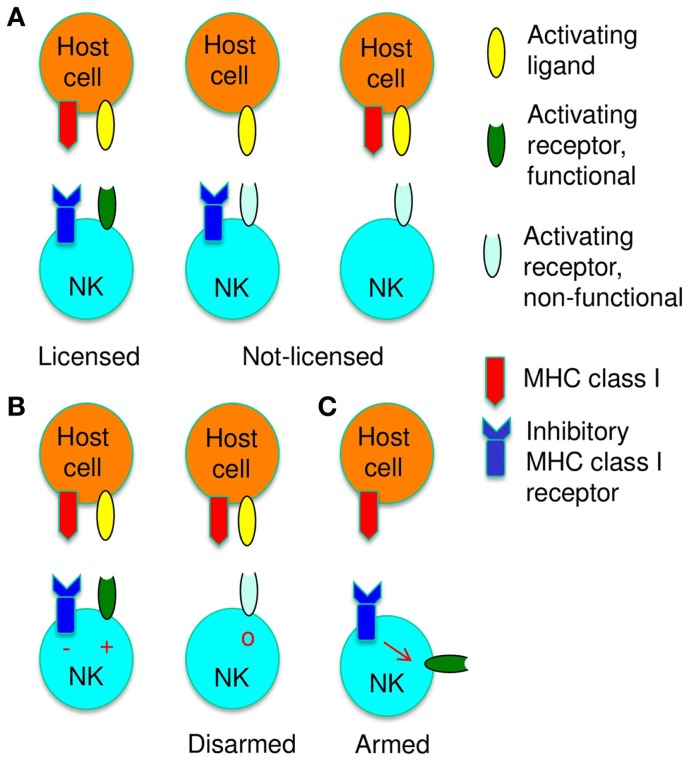
**Functional adaptation of NK cells to self-MHC class I is shown**. **(A)** NK cells are licensed, i.e., their activation receptors respond efficiently to stimulation (dark green) when NK cells can bind MHC class I. When the host lacks MHC class I molecules (middle) or when NK cells lack MHC class I receptors (right), activation receptors respond poorly to stimulation (light green), i.e., NK cells are not licensed. **(B)** Activation pathways are disarmed when NK cells are chronically stimulated in the absence of inhibitory signals from MHC class I receptors. **(C)** Signals from MHC class I receptors arm NK cells, i.e., instruct NK cell activation pathways to become responsive.

Optimal NK cell responses depend on an adaptive process, which adjusts NK cells functionally and phenotypically to the presence of MHC class I molecules. This process is globally referred to as NK cell education. Activation receptors on NK cells, which can recognize MHC class I, respond efficiently to stimulation, while NK cells, which cannot recognize MHC class I, respond poorly (19–22) (Figure 1A). NK cell licensing is used to describe the MHC class I-dependent change in the responsiveness of activation receptors (22). Licensing enables NK cells to respond to occasional aberrant cells that have lost MHC class I molecules (missing-self recognition). NK cell education is also associated with phenotypic changes, which include modifications of the repertoire of inhibitory receptors specific for MHC class I molecules (23, 24). However, these repertoire changes are subtle and their significance is uncertain, especially since they do not ensure that each NK cell can recognize MHC class I. Progress in the mechanistic understanding of NK cell education is hampered by a lack of cell surface markers to identify educated NK cells. Even though it is generally assumed that education is a developmental process, it is not known at which stage of development or maturation NK cells are educated.

KEY CONCEPT 1. NK cell education Education refers to all phenotypic and functional changes in NK cells imposed by the expression of MHC class I molecules.

KEY CONCEPT 2. NK cell licensing Licensing refers to changes in the function of activating NK cell receptors that depend on the recognition of MHC class I molecules by inhibitory NK cell receptors.

## Disarming NK Cells

One possible mechanism to explain NK cell education is that activation receptors become by default responsive to stimulation at some point during NK cell development. Acquisition of an inhibitory receptor specific for self-MHC class I would neutralize activation signaling and this would keep the activation pathway competent to respond. If NK cells cannot bind MHC class I, persistent stimulation would eventually disarm all activation pathways (NK cell disarming) (25) (Figure 1B), perhaps similar to the chronic activation described above. However, the relevant receptors, which activate NK cells in response to constitutively expressed self-ligands, are incompletely defined, even though SLAM family receptors explain in part the preferential reactivity of NK cells to normal hematopoietic cells (26).

KEY CONCEPT 3. NK cell disarming Disarming refers to a possible mechanism to explain licensing: Chronic stimulation of NK cells in the absence of MHC class I-dependent inhibition results in a reduced responsiveness of activating NK cell receptors.

This model is supported by the analysis of mixed bone marrow chimeras (27), MHC class I mosaic mice (28), and the adoptive transfer of NK cells from MHC class I-sufficient into MHC-deficient recipient mice (29). In all cases, NK cell function is dominantly impaired, when a significant fraction of the host cells lacks inhibitory MHC class I ligands. The transfer experiments show that mature NK cells are disarmed when transferred into an MHC class I-deficient host, indicating that disarming is not necessarily a developmentally regulated process.

The defining feature of this model is that the factors needed to inhibit NK cells are at the same time essential for NK cell licensing. MHC class I receptors cannot transduce inhibitory signals when their cytoplasmic ITIM (immune receptor tyrosine-based inhibition motifs) is mutated. Consistent with the above notion, such receptors do not license NK cells (22, 30, 31). Inhibitory signals are further transduced via the recruitment and activation of the Src homology 2 domain-containing phosphatase 1 (SHP-1). While NK cell licensing was dependent on SHP-1 function in some studies (27, 32) it was not necessary in others (22, 33). The conditional deletion of SHP-1 in NK cells will be essential to clarify this issue. Disarming as the unique explanation for NK cell licensing was called into question based on mice expressing a variant of the murine Ly49A receptor, in which the flexible stalk region was replaced with a rigid stalk derived from the CD72 receptor (see below) (30, 31). The variant receptor readily inhibited NK cell-mediated lysis of target cells. However, these NK cells did not mediate missing-self recognition and their NK1.1 activation receptor was not licensed (30, 31). These results suggest that the inhibitory function of an MHC class I receptor is not necessarily sufficient for NK cell licensing.

## Arming NK Cells

An alternative mechanism for licensing is that MHC class I recognition instructs NK cell function. Accordingly, NK cell activation receptors would by default respond poorly to stimulation. The engagement of MHC class I receptors would be needed to render activation receptors responsive (NK cell arming) (25)(Figure 1C). Indirect support for this model came from the study of the aforementioned Ly49A variant, which suggested that the inhibitory function of the MHC class I receptor did not suffice to license NK cells (30, 31). The notion that ITIM-bearing Ly49 family member can perform functions in addition to inhibition is supported by the analysis of Ly49Q. This receptor enhanced, rather than inhibited, TLR9-mediated signaling events in plasmacytoid dendritic cells. The positive effect depended on the Ly49Q ITIM, but did not require SHP-1, SHP-2, or SHIP phosphatase activity (34). Thus ITIM-bearing Ly49 receptors can improve cellular activation. Finally, leukemia patients receiving an allogeneic stem cell transplant may have donor-derived NK cells that exert reactivity against residual leukemic cells. This depends on KIR on donor-derived NK cells that can recognize HLA class I of the donor but not of the host (KIR ligand mismatch) (35). These NK cells thus appear to be licensed by the presence of donor HLA class I (36). Similarly, an improved NK cell control-mediated of murine cytomegalovirus infection after allogeneic stem cell transplantation was dependent on the MHC class I of the donor rather than that of the host (37). Based on the disarming model, the absence of MHC class I ligand from a fraction of cells is expected to dominantly induce NK cell dysfunction. The above transplantation settings provided evidence that the presence of donor-type MHC class I molecules instructed NK cell function, consistent with arming.

KEY CONCEPT 4. NK cell arming Arming provides an alternative mechanistic explanation for licensing. MHC class I recognition instructs NK cells to render their activating receptors responsive to stimulation.

## NK Cell Education and the Role of MHC Class I Recognition in *trans* and *cis*

Murine Ly49 family receptors have the capacity to interact with MHC class I expressed on other cells (in *trans*) as well as on the NK cell itself (in *cis*) (38). Even though a significant number of additional cell surface receptors can bind ligand expressed in the plane of the same membrane (39), there is currently no evidence that KIR or CD94/NKG2A receptors are influenced by MHC class I expression *in cis*. For Ly49 receptors, combined structural and functional analyses showed that *trans* and *cis* interactions are mediated by two distinct conformations of Ly49 receptors (40). The existence of distinct Ly49A conformations on the surface of live cells was confirmed using a FRET-based approach (41). A question arising from these studies was whether *cis* interaction contributed to NK cell education. The aforementioned Ly49A variant was actually designed to allow *trans* interaction while preventing *cis* interaction. This was achieved by replacing the flexible Ly49A stalk with a rigid stalk from the CD72 molecules, thus forcing the ligand binding domains away from the NK cell membrane. As mentioned above, this receptor inhibited the function of cytokine stimulated NK cells but failed to license NK cells. Thus *trans* interaction was not sufficient to license NK cells, indicating that *cis* interaction played a role (31). This issue was addressed further using a shortened Ly49A receptor, which could no longer inhibit NK cells (absence of functional *trans* binding) but which retained the ability to bind in *cis*. This receptor also failed to license NK cells (30). Thus *cis* interaction alone was also not sufficient to license NK cells, raising the possibility that both *cis* and *trans* binding were required. The same conclusion was drawn based on an independent approach, in which the H-2D^d^ ligand of the Ly49A receptor was switched off in a cell type specific fashion. The deletion of H-2D^d^ selectively from NK cells (in *cis*) prevented NK cell-mediated missing-self recognition. Missing-self recognition was also impaired when H-2D^d^ was deleted selectively from T cells, i.e., in a situation where *trans* interactions with a specific cell type was abrogated (30). These data were consistent with the analysis of the modified Ly49A receptors and suggested that both *cis* and *trans* recognition of MHC class I molecules played a role for licensing when NK cells developed at steady state.

On the other hand, a role of *cis* interaction for NK cell licensing was called into question based on adoptive transfer experiments (29, 42) and the induction of MHC class I expression *in vivo* (43). Following adoptive transfer of MHC class I-deficient NK cells into MHC class I-sufficient mice, NK cells readily acquired improved function. Thus, the expression of endogenous *MHC class I* genes by NK cells was not needed to license NK cells. However, MHC class I-deficient NK cells are known to acquire significant quantities of MHC class I molecules from surrounding cells via their Ly49 receptors (44, 45). It was thus possible that licensing occurred via *cis* interactions with the acquired MHC class I molecules (42). When H-2D^d^ was selectively deleted from NK cells, Ly49A+ NK cells acquired H-2D^d^ from surrounding cells, however, licensing was not observed (30). Thus *trans* recognition seems sufficient to improve function following an acute exposure of peripheral NK cells to MHC class I. One possibility to explain the discrepancy to the findings described above may be that the developmental stage determines how NK cells respond to MHC class I encounter. Consistent with this possibility, modifications of the MHC class I receptor repertoire are observed when NK cells develop at steady state (23, 24) but are not evident following the adoptive transfer of MHC class I-deficient NK cells into MHC class I-sufficient hosts (42) or the acute induction of MHC class I expression *in vivo* using a tetracycline regulated expression system (43).

## A Sequential Arming/Disarming Model for NK Cell Education

**Figure 2:**
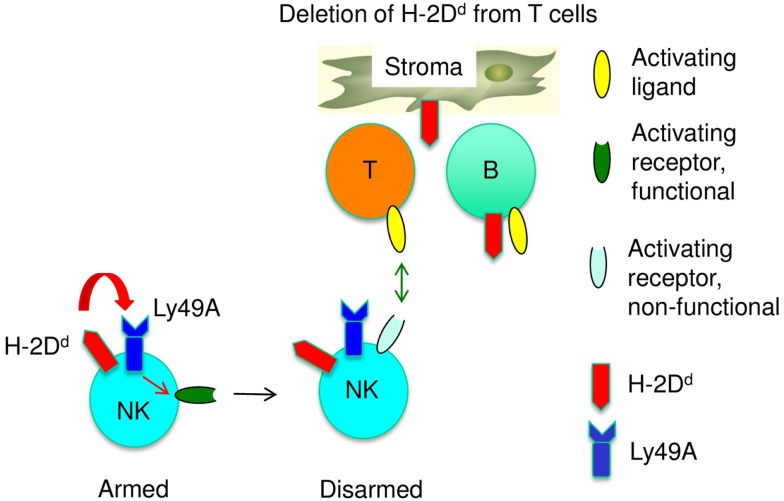
**A sequential arming/disarming model for the adaptation of immature NK cells to self-MHC class I is shown**. The co-expression of Ly49A and H-2Dd by NK cells, and thus their interaction in *cis*, renders NK cell activation receptors functional (arming). The absence of H-2Dd from other cells in trans (e.g., T cells) results in chronic NK cell stimulation by T cells, which will eventually disarm activation pathways.

To account for a role of both *cis* and *trans* recognition for NK cell licensing of immature NK cells, we propose a sequential arming/disarming model for NK cell education. This model suggests that *cis* interactions instruct NK cell activation pathways and that *trans* interactions provide inhibitory signals, which prevent disarming (Figure 2). If so, the lack of *cis* and *trans* recognition should have distinct effects on NK cells. When MHC class I was absent form NK cells (*cis*), NK cells were not licensed. In contrast, NK cells appeared licensed, i.e., the NK1.1 activation receptor was functional, when MHC class I was absent from T cells (*trans*). A similar discrepancy between licensing and missing-self reactivity was reported in a *CD1* knock out mouse strain. However, the reason for the defect is currently unknown, since it is independent of the targeted mutation (46). While the basis for this novel type of NK cell tolerance needs to be explored, impaired missing-self recognition induced by the absence of MHC class I in *cis* or from some cells in *trans* seems to be based on different mechanisms.

KEY CONCEPT 5. Sequential NK cell arming and disarming We propose that MHC class I recognition during NK cell development first instructs NK cells to render their activating receptors responsive to stimulation (arming) and then prevents the chronic activation of NK cells, which would reduce the responsiveness of activating NK cell receptors (disarming).

An additional aspect of NK cell education is the modification of the repertoire of MHC class I receptors (23, 24, 47). The expression of a wild type Ly49A transgene in H-2D^d^ mice is known to induce significant changes in the endogenous MHC class I receptor repertoire. In contrast, repertoire changes were limited when H-2D^d^ was deleted from NK cells (*cis*), when Ly49A could only bind in *trans* and when the Ly49A ITIM was mutated. Conversely, repertoire changes were substantial when MHC class I was deleted from T cells, i.e., when Ly49A could bind in *cis* but not to certain cells *in trans*. Thus, the repertoire was modified whenever MHC class I was recognized in *cis* and when the ITIM was intact. However, when Ly49A could not also productively or continuously interact with MHC class I in *trans*, NK cell function was not improved. Thus repertoire changes chiefly depended on *cis* interaction while improved NK cell function required both *cis* and *trans* interactions. Accordingly, we propose that *cis* interaction would instruct NK cell activation receptors to become responsive. As a consequence, NK cells would modify the MHC class I receptor repertoire and *trans* interactions would be needed to maintain the functionality of NK cell activation pathways. If MHC class I receptors interact only in *cis*, but cannot not productively (shortened Ly49A variant) or continuously (T cell specific H-2D^d^ deletion) interact in *trans*, NK cells are not protected from the adverse effects of chronic stimulation and activation pathways would be disarmed (Figure 2). If MHC class I receptors interact only in *trans*, NK cell activation receptors are not instructed and the MHC class I receptor repertoire does not adapt.

## Concluding Remarks

Natural killer cells functionally adapt to the presence of MHC class I molecules in order to detect cells that lack this key identifier of “normal self.” We propose a model in which MHC class I specific receptors have two roles, i.e., to first establish and then to maintain NK cell function. During NK cell development, Ly49 receptors mediate these two functions via *cis* and *trans* interactions, respectively. MHC class I receptors, for which there is no evidence for *cis* binding, which includes CD94/NKG2A or KIR, may also educate NK cells in a sequential fashion but perhaps via distinct types of signals based on *trans* interactions. In addition to directly address this issue, it will be important to determine whether the developmental or maturational stage differentially impacts the functional adaptation of NK cells to the presence and absence of MHC class I. Elucidating the molecular mechanisms of how NK cells adapt, may allow the identification of therapeutic targets that prevent NK cells from adapting to their environment, including growing tumors.

## Conflict of Interest Statement

The authors declare that the research was conducted in the absence of any commercial or financial relationships that could be construed as a potential conflict of interest.
